# Rural settlements dynamics and the prospects of densification strategy in rural Bangladesh

**DOI:** 10.1186/s40064-016-1883-4

**Published:** 2016-03-01

**Authors:** A. F. M. Ashraful Alam, Rumana Asad, Md. Enamul Kabir

**Affiliations:** Department of Geography and Planning, Macquarie University, Sydney, NSW 2109 Australia; Architecture Discipline, Khulna University, Khulna, 9208 Bangladesh; Forestry and Wood Technology Discipline, Khulna University, Khulna, 9208 Bangladesh; Center for Southeast Asian Studies, Kyoto University, 46 Shimoadachi-cho, Yoshida Sakyo-ku, Kyoto, 606–8501 Japan

**Keywords:** Densification, Rural settlements dynamics, Bangladesh

## Abstract

Given the year on year decrease of rural farmland and various forms of land degradation through the intrusion of non-farm land uses, the government of Bangladesh has drafted the agrarian reform strategies, primarily to protect the agricultural land from encroachment, conversion, and indiscriminate use. The draft Agricultural Land Protection and Land Use Bill since its inception in 2011 is facing serious uncertainties of implementation due to its borrowed nature from the developed contexts and inadequacy to recognize the local complexities. With a particular focus on the densification component of the draft bill, a semester-long design studio was conducted in consultation with the existing villagers to explore the practicability of the draft bill in the villages of Tetultala and Chhoygharia in the south-western coastal Bangladesh. The findings from the two villages hint that in Bangladesh, the unique and evolving nature of rural settlements dynamics that are disintegrating the rural society from farming practices and the farmland, thereby, unsettling the traditional village-morphology. The settlements dynamics vary from those of the western context; hence, there is an emerging need to build locally situated knowledge towards a feasible rural land reform.

## Background

The rural farmland is in continuous decay as the unique and vernacular human-land interaction of Southeast Asia (the predominantly rural continent of the last century) is contested through urbanization and related development aggravations. Bangladesh is no exception with two-third of the populations lived in villages in 2014 (FAO 2015). 41.54 % of the economically active population is engaged in agriculture contributing 20 % to the country’s total GDP. In the absence of any stringent planning and land use control for rural areas, the agricultural lands are now dotted with commercial, residential, industrial, and other inappropriate uses. Together they hint deterioration in the traditional rural morphology resulting in the decline of agro-based income and productivity (Quasem 2011).

As the unplanned encroachment of rural land seems to have arrived at crossroads, recently, concerns have been raised not only to ensure the region’s food security but also for the sustenance of agricultural practices of the large rural demography in retaining the traditional village morphology. Urgent response is needed through integrated planning and land use controls to prevent any abusive disposal of rural farmland. In response, the government of Bangladesh has demonstrated the *Draft Agricultural Land Protection and Land Use Bill* in 2011 (BSS 2011). Under the proposed law, non-farm occupation (i.e., construction of housing, industry, shrimp hatchery, brickfield) of arable land is strictly prohibited with necessary enforcement initiatives.^1^ The bill proposes the mandatory confinement of non-farm land uses to the existing non-farm lands. In addition, the draft bill has explicitly discouraged any alteration of the existing landscapes including rivers, canals and marshlands that should be exclusively protected for fisheries and rainwater retention. Importance has been put on the reclamation of the illegally captivated farmlands including many watersheds to their earlier states.

Although for a while the government shied away to implement the bill (Rahman 2013), recently concerns have been put to initiate a separate land-zoning map (Hasan 2015; Karim 2015). However, these initiatives seem to be incongruent to the organically evolved countryside context of Bangladesh. Given the fact that the government’s intention to intensify non-farm land uses to non-farm lands is a timely initiative, the bill inevitably points towards the notion of density-control in rural areas as the future major policy instrument. Densification of the rural non-farm settlements in order to protect agricultural land was made explicit throughout the draft document. However, historically rural settlements of Bangladesh did neither undergo such radical and formal planning intervention, nor there has been adequate institutional capacity.

Amidst the uncertainty of policy intervention, in recent years, the rural agrarian socio-spatial dynamics of Bangladesh has changed. There has been increased dependency of the rural population to urban centers for livelihood opportunities,^2^ unequal distribution of land-based resources,^3^ and instability to the rural environment (i.e., reducing wetland, aquatic biodiversity and dwindling natural forest).^4^ With relatively small landholdings of 25 decimal per capita out of which only 15 decimal are left out for agricultural use (Barakat et al. 2007), the occupation of farming does not seem much lucrative fro the villagers. The paper begins with arguing that any policy measure (such as, densification) relating to rural land reform needs to be compatible with the present day socio-spatial complexities and demands of the countryside. Therefore, the paper aims to understand the existing rural settlements dynamics of Bangladesh in order to explore the prospects of the proposed agricultural land protection strategies with specific focus on the draft bill’s densification component.

### Framing ‘densification’ in the draft bill of 2011

Historically land regularization was never central in the rural planning (if existed at all) and policy context of Bangladesh. Only the 1950 State Acquisition and Tenancy Act and the 2001 National Land Use Policy feebly touched upon the land reform strategies by acknowledging the restriction of agricultural land against non-farm purposes. However, there remained plenty of room for relaxation, the restrictions were largely ineffective (LANDac 2012). There was still plenty left to consume from the rural land bank, therefore, the exchange value of cropland also remained low. Until recently, the economic importance of rural land escalated because of the urban expansion of the major cities occupying village land and escalating the demand of space for industries and other non-farm sectors. Considering the alarming rate of cropland shrinkage and the emerging tension between agricultural and non-farm dynamics, the past scanty policy measures are ineffective and they require major turnaround to the emerging problem-focus of limited farmland supply.

The *Draft Agricultural Land Protection and Land Use Bill* of 2011 is a response towards the recent ineffective policy context. It aims to bring villages under prudent ‘zoning principle’ to stop any illegal captivation of agricultural land, as often the politically and financially powerful grabbers hold large ceiling of agricultural land for non-farm development. The bill demonstrates a strong determination to restrict the existing agricultural land only for cultivation under any circumstance. Under the resolution, all the ‘*Khas*’ lands must be retained for cultivation only. Existing landscape elements (i.e., hills, water sheds and woods, etc.) are advised to retain intact and non-convertible to other usages under any circumstances. Apart from the protection measures, the bill principally encourages the compact development strategies for the existing non-farm lands to ensure maximum utilization and minimum wastage of spaces. Acquisition of land for infrastructure is advised to keep the minimum. Most importantly, ‘vertical extension’ of rural dwelling spaces has been advised as a priority measure for new built-forms conforming local development plan, rural characteristics, and the environment.

The proposal for a systematic compaction of the non-farm uses to the non-farm lands in order to keep the farmlands free from non-farm occupation indicates a major paradigm shift. The increase in development intensity through vertical extension of built-forms in non-farm land parcels implies that there would be increasing number of population in the homestead land. The intention to increase density in the homestead land would eventually demand alteration in the traditional rural resource management system. Within the limited resource based third world villages the proposal of a compact rural form seems apparently incompatible to the highly informal and relatively disperse rural settlements systems. In addition, while density design to achieve a compact and sustainable urban form is questioned for traffic congestion, air pollution, unhealthy living and psychological discomfort (Burton et al. 1996; Dieleman and Wegener 2004; Gordon and Richardson 1997; Jenks and Burgess 2000; Jenks et al. 1996; Morrison 1998; Neuman 2005), its implication in the third world rural settlements seems to be far from being consistent and demands rigorous scrutiny prior to real time application.

### Framing the research

**Fig. 1:**
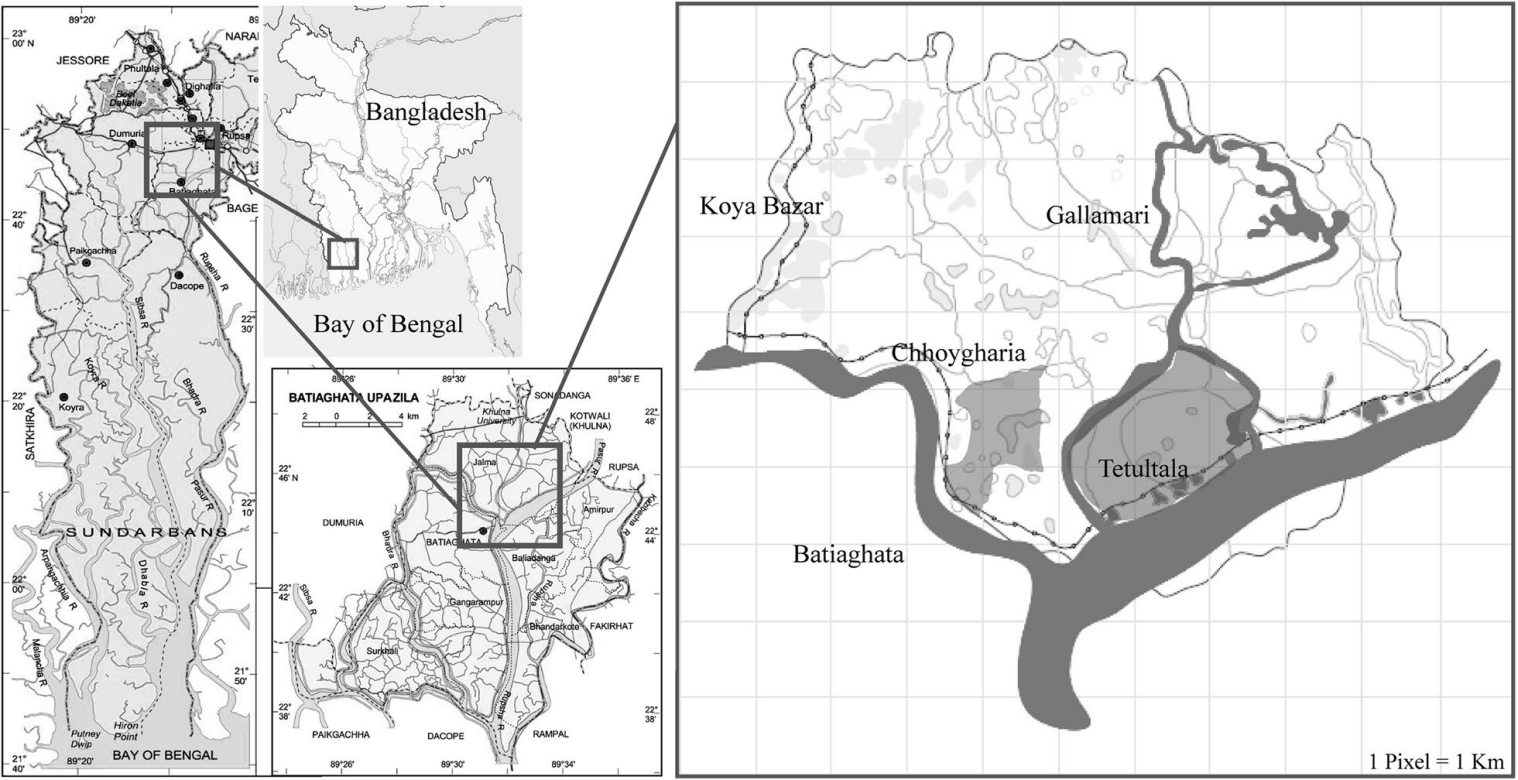
Location map of Tetultala and Chhoygharia villages in Bangladesh. *Source* authors extracted from google map

The study took two representative villages named Tetultala and Chhoygharia (Fig. 1) of Batiaghata Upazilla in the southwestern coastal district of Khulna Bangladesh. A semester-long urban design studio was conducted. Four groups each comprising of six undergraduate students of Architecture Discipline in Khulna University were assigned to develop future scenarios (after 50 years) of the villages. The scenarios were developed based on the hypothetical application of the densification concept. Over a period of 13 weeks, with close consultations with the existing villagers, the scenarios were matured based on the detail interpretation of the draft bill, desktop research, existing strength-weakness-opportunity-threat analysis and an extensive mapping exercise (of physical land use, infrastructure, transportation, water-land ratio, land ownership patterns, local administration boundaries) and feedbacks from the experts in successive phases.

**Fig. 2:**
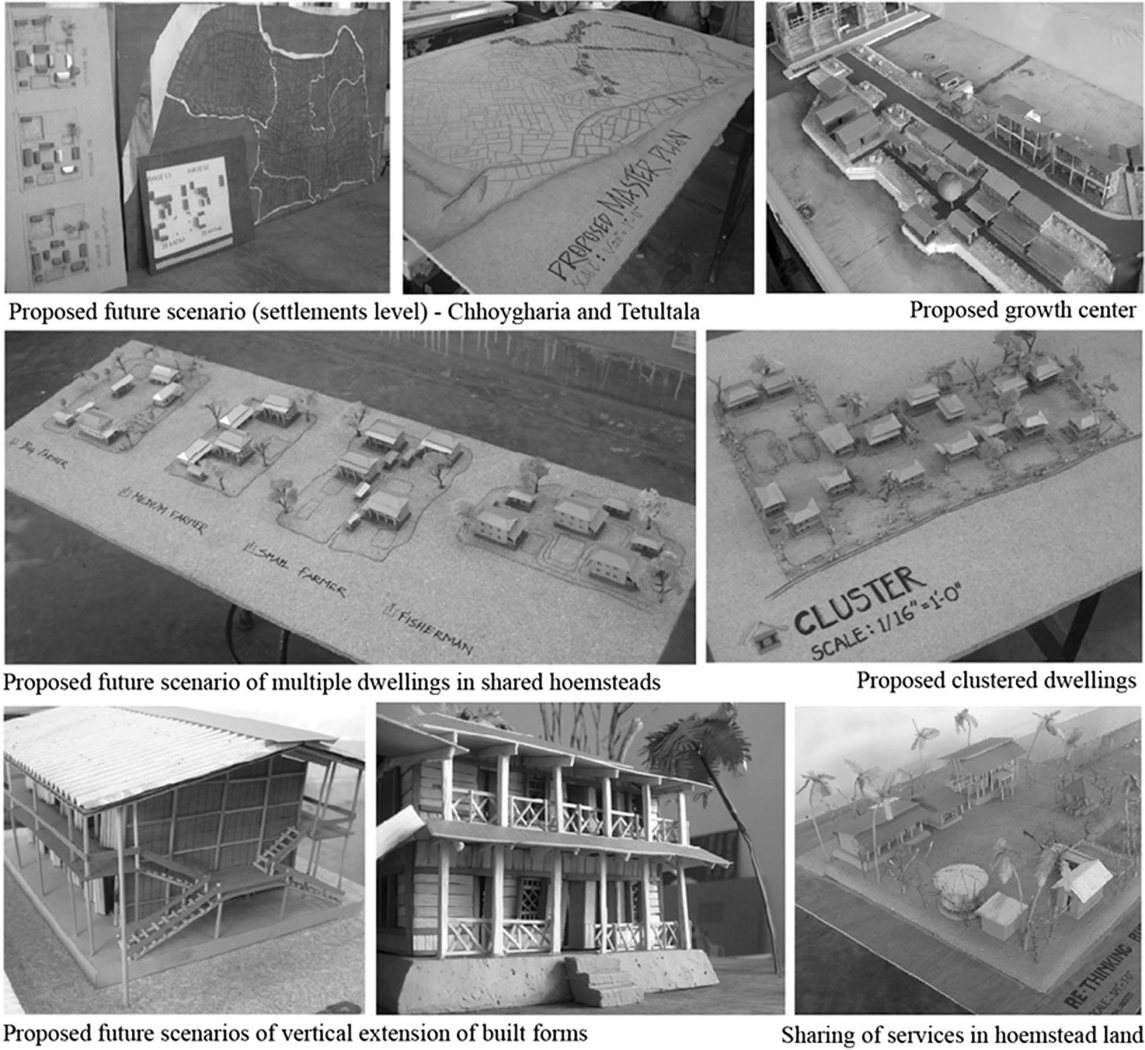
Design outcome of the hypothetical scenarios of Tetultala and Chhoygharia. *Source* studio works conducted by authors and documented in Hossain (2013, pp 106–111)

The design studio experimented on different alternatives for the two villages as per the densification intent of the 2011 draft bill. As presented in the Fig. 2, the design was conceived in three phases. Firstly, at the settlement level the farmland, the existing underutilized or illegally occupied government land and the other natural resource base (such as, forest land and water bodies) were identified that eventually helped to clearly demarcate the non-farmlands for future densification. Then, for the non-farm land, as per the existing demographic trend the future population density after 50 years was calculated. Based on the projected density, the existing homesteads were proposed in forms of different cluster organizations of multiple dwellings. Finally, vertical extensions of the built-forms accommodating different non-farm land uses (such as, residential built forms, commercial growth centers, etc.) were visualized.

While the detail discussion of the design outcome is beyond the scope of this present paper, the leanings from the studio exercise create an entry point to the complex socio-spatial dynamics of the present day villages in Bangladesh. The following sections of the paper present an interpretive analysis on the critical leanings from the studio projects based on the feedback from the villagers on the proposed designs. Given that the strategy of densification is a borrowed concept from the developed context and acknowledging that villages and non-urban areas have long been neglected areas in urban studies in developing contexts, the paper discusses relevant literature from developed context to draw on the incommensurability of the rural settlements dynamics between the villages in developed contexts and those of Bangladesh. Finally, the prospects of rural settlements dynamics are discussed.

### Lessons learnt from the hypothetical scenarios

Empirical evidence confirms that the villages of Tetultala and Chhoygharia and their ongoing structural and spatial transformations are representative to the villages throughout Bangladesh. There remains nothing pristine about villages in Bangladesh as they are more or less affected by the urban. Historically the population of the two study villages lived on agriculture. 1980s onwards, the expansion of Khulna city to its hinterland and greater rural–urban accessibility instigated rural–urban continuum resulting to a steady conversion of agricultural land to non-farm uses. According to the villagers, due to the heightening aspiration for non-farm jobs in town centers over traditional farming, the rural farmlands are gradually being disposed off to the non-local urbanites. These absentee urbanites now hold of a large share or rural land (refer to Table 1). Furthermore, increasing literacy rate, empowerment of women through government initiatives and NGOs’ supports, availability of less laborious non-farm jobs, land price hike up to 100 times-all transformed these two villages spatially and socially during last 10 years.

**Table 1:** Comparative profile of Tetultala and Chhoygharia villages

	Tetultala	Chhoygharia
Area in sq. kilometer	3.96	1.8
Population size by number of people	2740	3000
Number of families	383	430
Population density (persons per acre)	3	6
Male/female ratio (%)	47/53	49/51
Literacy (%)	95	73
Change in no. of attendance to tertiary education in last 15 years	0-13	6-52
Population engaged in farming (%)	90	83
Conversion of farmland to non-farm use (%)	13.5 % in 25 years	10 % in 20 years
Illegal occupation of water channels (%)	90	30
Amount of ‘Khas’ land (%)	32	38
Land owned by outsiders/non-villagers (%)	62	70
Land price change during 2009–2014 (times)	10–100	10–60

Source authors’ field survey as part of the studio project in 2013

Analysis of the draft bill unfolded the densification strategy having two intrinsic motives, the first one, the compaction of non-farm land uses to non-farm lands and secondly the protection, reclamation and expansion of farmlands at any cost. Despite the hypothetical nature of the studio project, as the design was presented to the villagers, the first objective of consolidating the non-farm uses to the existing non-farm land was found unrealistic due to the demand for an uneconomic infrastructure provision in a relatively low-income, organically evolved and dispersed existing settlements. The existing density of 3–6 persons per acre (Table 1) was too low to generate mass threshold to initiate a feasible compact rural form.

Secondly, during the consultation phase, the proposed vertical extension of homestead built-forms against the traditional horizontal nature of residential spaces (as shown in Fig. 2) was opposed and found unrealistic by the villagers. They also suspected that the proposed amalgamation of multiple residential lots to large shared plots would heighten the complexity of multiple land ownership. In addition, the increasing loss of traditional lifestyle, increased urban influence and interest in nucleated family lives would be critical to the acceptability of the sharing of resources in the homestead. Feedback from the villagers also revealed that vertical extension of built forms would create new winners and losers as the competition among stakeholders of one single homestead would be unavoidable; nobody had wanted to leave the ground level and ‘live in the sky.’

According to Jenks (2000), the sustainability benefit from compact development can be greatly enhanced when a shared attitude within the social structure and a mixed nature of spatial development are ensured. Given the increasing heterogeneity of the contemporary village demography (as shown in Table 1), i.e., increasing number of non-farmer occupancy delving inequality stakeholders; a community based cooperative lifestyle and social structure sounds difficult to achieve. Furthermore, excessive dependency on the nearest urban center for jobs and other opportunities were evident in both the villages. Therefore, in the design feedback stage the participant villagers rejected the proposals of the mixed land use solutions regarding the village growth centers. One of the villagers expressed, ‘what the cities offer probably can never be substituted by a village growth center.’

The second intrinsic motive of the densification strategy, the protection, reclamation and expansion of rural farmland was largely found unrealistic by the villagers due to the increasing diversity of stakeholders in the case study villages. Discussions revealed that there were as many interests on land as there were as many types of occupants. In particular there is an evolving nature of land speculation and politics surrounding land in which the absentee urban landlords, local politicians, the remaining few large village farmers and the land-grabbers play a dominant role in land use decisions (e.g. industries, urban types of housing development). Prevalence of these multiple socio-spatial settlements dynamics holds back in achieving any common consensus towards cooperative farming, utilizing shared farming technologies or protection of the agricultural land.

### Rural settlements dynamics: Perspectives from the outside

Rural settlements present a spatial facade to the aspatial structures and processes (Cloke and Hanrahan 1984), where the physical land is psychologically embedded with many different yet deeply held meanings (Gilg 2002) for unique communities (Akgün et al. 2011). Given the processual nature of the human-land interaction in the rural system (Zhou et al. 2010) accommodating multi-level, multi-actor and multi-facetted socio-political struggles over time and place (van der Ploeg et al. 2002) there is no universally accepted theory that explains the rural and predicts its future (Clark et al. 1997; Nooij 1997; Singh 2009). Within such volatility the rural settlements dynamics should incorporate a more context-specific knowledge that adequately explains the historicity of the spatial and aspatial transformations of the countryside.

In the past, the term ‘rural’ was often determined by its indicators of ‘visible ill’ (Bowler et al. 2002) such as, lack of density to provide efficient services (Moseley 1979), inadequate employment opportunities, selective depopulation resulting from rural–urban continuum and intra-class conflict of vulnerable groups (Cloke and Thrift 1987). Historically, these attributes left enough room for agriculture to play freely in the rural social, cultural and political system, until the point, settlements started to experience ripples of modernism (van der Ploeg 1995a). Through realignment of the traditional production system with associated technological fixes (Leichenko and O’Brien 2002) as a response to the extra-local demands (Depoele 1996), the countryside became although overcame the historic illness but entered into more complex agriculture-industry-market landscapes.

Until the 1980s modernization of villages by linking industries with agriculture remained largely unacceptable (Piore and Sabel 1984) because of the continuing decline of agricultural practices across Europe and as an aftermath the rising insecurity regarding landscape, nature, and environment (Knickel 1990; Mannion 1996). As a result, conservation and economic development often supplemented as mutually inclusive dynamics to the extent where rural land-use planning and management had been able to retain environmental values (Bowler et al. 2002; van Lier 1998). However, over time modernism’s exogenous dynamics (van der Ploeg 1995b, 2000; van der Ploeg and Saccomandi 1995) inevitably took place by linking agricultural and non-farm rural activities. Eventually, local participation and resources including labor force, knowledge and links between production and consumption practices started to emerge as the endogenous dynamics (Lowe et al. 1995; Volker 1997).

Modernization trickled down to the villages and established a very complex ‘ambiguous interdependency’ with cities (Smithers et al. 2005). Technology replaced the traditional farming system (Curran and Storey 1993; Elbersen 2001). Increasing mobility of people, goods and information started opening up the countryside to new uses (Munton 1995; Murdoch and Marsden 1995). The changing role of agriculture with the transformed landscapes, depopulation, extinction of the traditional peasant society and the cascading effects of such changes surfaced as drawbacks (Varga and Varga 2008). Villages replaced their earlier distant role and appeared as the emerging ‘hinterland’ of cities ensuring the supply food (Marsden et al. 1993). They also offered amenities and other new economic activities (Brown and Grilliard 1981; Chisholm 2007 signaling the emergence of a new breed of rural settlements dynamics.

To address these contemporaneous changes in villages of Europe, Marsden (2003, pp 4, 12) suggests three interrelated, overlapping and competing dynamics displaying their own socio-spatial expressions and the dynamics need to be balanced to retain rural sustainability. The first one directs to the agro-industrial linkages referring to standard farmed products including the complex supply chain provisioned through technological fixes (Marsden et al. 2002; Renting et al. 2003). While this hints an intelligent management of rural spaces to link agricultural and non-agricultural structures (Allanson et al. 1995), the post-productivist one refers to the rural land as a development space (Marsden 1995, 1998). These two dynamics transform the rural spatial facade, where the ‘vertical’ one links the rural spaces into the agro-food sector and the ‘horizontal’ network links with the non-agricultural processes of economic change (Clout 1993; Lowe et al. 1993; Murdoch 2000). Due to their intensive and exploitative nature, the dynamics create imbalance in the rural aspatial facades through social exclusion of the marginal. Eventually, the historic nature, local communities and culture (Ruda 1998) get endangered. Marsden’s third dynamics refers to softer development interventions in form of sustainable livelihoods, tenure security, diversity of income new institutional arrangements and policy for economic development without outreaching the carrying capacity of the settlements (Dalal-Clayton et al. 2003). Gallent et al. (2008) also refers to similar dynamics within the framework of rural economy more aligned to the market, environmental changes and good governance through community development.

### Rural settlements dynamics: perspectives from Bangladesh

The age-old pastoral lifestyle in Bangladesh is now fragmenting apart although a while ago they were literally self-sufficient as all the requirements of living could be met from within the rural limit. Global values exert powerful economic and social influences on the traditional limits of the countryside. The changes in rural demography, rural–urban continuum, technologies in agriculture including the extended supply chain and market linkages facilitating powerful actors and cornering the marginal to the peripheries—they all induce diverse dynamics and continuously reconfigure the socio-spatial structure. Increasing urbanization transforms the rural agricultural land to a commodity signaling the new regime of post-agro land economy. Above all, the social-cultural changes through flow of information and various forms of development aids are trickling to the farthest corners of the countryside, redefining the rural resources and open up new non-farm livelihood opportunities. Therefore, farming and farmland as central to rural values are contested.

Diminishing interest in farming encourages unplanned and dissonant changes on rural land affecting year-on-year loss of cultivable land.^5^ It eventually reduces the farmland share of households^6^ thereby reducing agro-economic activity^7^ and household income.^8^ High dependency on technology is replacing the production system, the demand for high cropping intensity^9^ in relatively limited land and increasing salinity of sub-soil due to sea level rise and climate change^10^ yielded the rural system-change leaving a large number of marginal farmers incompetent. The increased demand for commodity (and exotic) crops in the global market has exerted ambiguous occupation of the land previously reserved for the staple food-crops.^11^ Modernization of agriculture only benefits a small number of large stakeholders, however, its unaffordability leads the majority common farmers eventually losing both interest and competency in farming. They are forced to dispose the land off through various channels. The rise of intermediaries extracting larger profit than the actual producer in absence of favorable government policy only accelerates the process and extent of the land disposal.

Eventually, a new non-farm land economy proliferates with the diminishing acceptance of agriculture as feasible economic practices for many. It systematically leads to devaluation of rural farmland and fosters land conversion giving way to non-farm uses through speculation, fragmentation and land-sale.^12^ The growing urban population triggers further thresholds stimulating the demand for residential land.^13^ It triggers the higher incidence of land transaction price (refer to Table 1), often in the hand of absentee landowners from cities- the ultimate symptom of a very active land market. The disposal of agricultural land through sale becomes a lucrative and fast cash incentive.^14^ Industries mushroom in the fringes due to the supply of cheap land and the new non-farm labor force. Increasing occupation of the ‘khas’ land by the landless villagers becomes visible.^15^ It is no surprise that the public waterways have a consistent history of being erased from the rural fringes through real-estate speculation.

Increasing accessibility to non-farm economic opportunities and growing number of nuclear family units imbue new dynamics in the rural spatial and aspatial structures (Adnan 1996; Ahmed 2006). Increased literacy rate,^16^ flow of remittances^17^, access to electricity,^18^ electronic media and information technology^19^ have created a new mix of winners and losers who further distance themselves from farming practices. The NGOs have a major contribution in rural Bangladesh to mobilize the women (Rozario 2002) and make them free from their previously unvalued labor at home and in the field (Afsar 2003). Growing landlessness leaves little room for them to remain confined within the domestic premise. They search for opportunities both near and far creating further disintegration in the agrarian lifestyle.

### The prospects of densification in rural Bangladesh

**Fig. 3:**
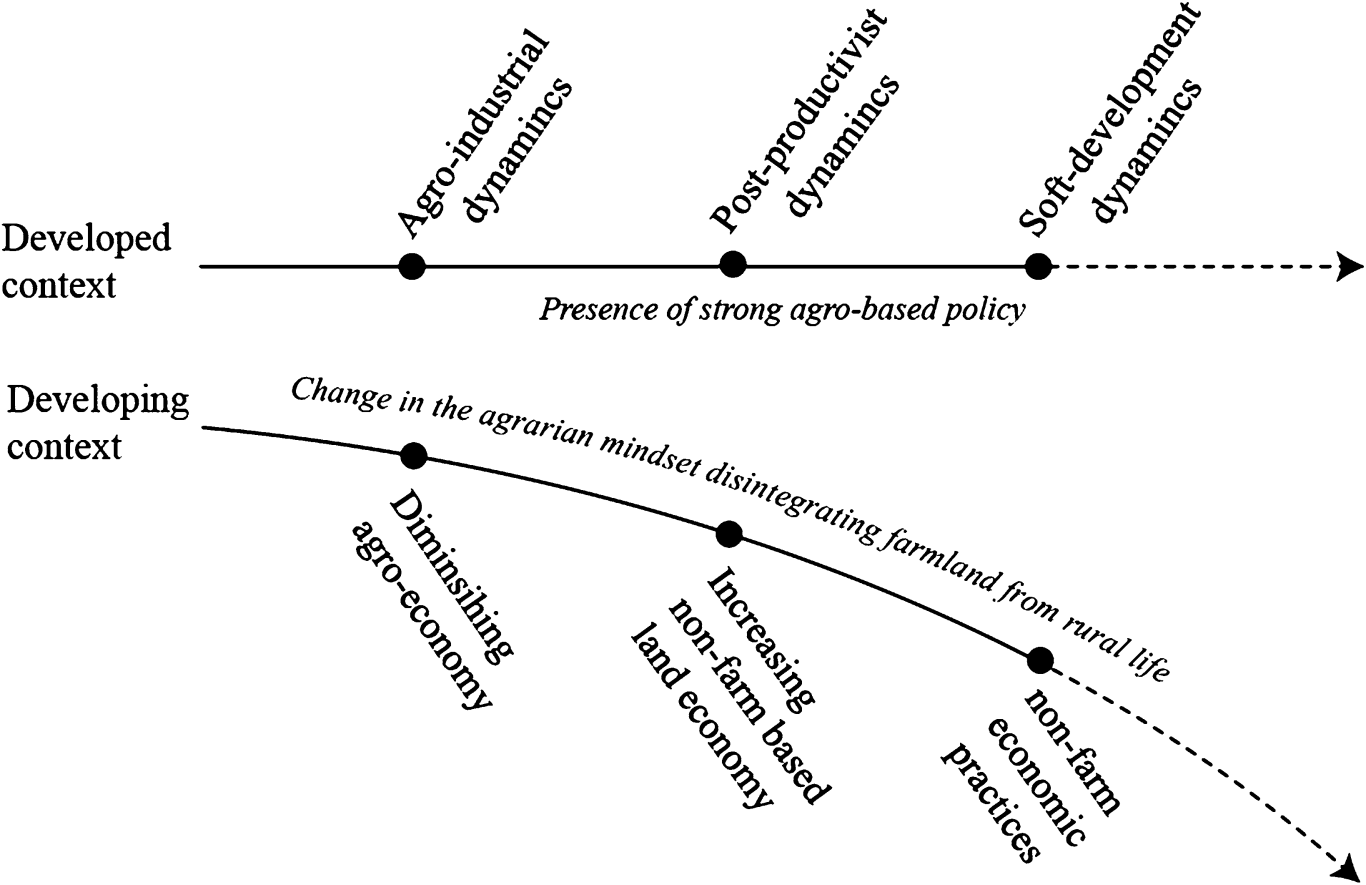
Differences in rural settlements dynamics between developed and developing context. *Source* authors

With all these socio-spatial transformations in the villages of Bangladesh the rural population is constantly distancing themselves from the agricultural practices, thereby from the rural land. Although the settlements dynamics are multi-faceted, multi-scalar and complex, a simplified comparison between the developed context and the developing context has been conceptualized in Fig. 3 to show the disintegration between traditional agricultural practice and the rural population.

Figure 3 explains that the spatial and structural format of villages in the first world may not able to explain the rural settlements of the third world. By the time the rural settlements in the West entered the post-productivist era through securing the balance among the horizontal and vertical links of agriculture and non-farm sectors, in contrast, the rural settlements in developing context are yet to experience any systematic modernization. Villages in Bangladesh still stand in stark contrast to the developed context in terms of demographic and resource distribution. However, globalization and the knowledge base of modern era simulate both the developed and developing societies and force to restructure the vernacular knit even of those most remote rural settlements. The rural Bangladesh also experiences the transformation of the primary agricultural production spaces to non-farm spaces of various sorts, however, in unregulated and informal manner. To deal with the non-specificities, the more contextual and southern perspectives informed by authentic settlements dynamics are long overdue to reinforce any spatial reform.

Fig. 3 shows that due to the presence of a heavily regulated agro-economic focus in the developed context an interdependent rural–urban formation proliferates. On the contrary, the diminishing interest in farming coupled with the rising non-farm land-economy and the non-farm livelihood opportunities subtly disintegrates the agricultural land from the center stage of the rural social conditions. Increasing exchange value of land as commodity proves the traditional farming practices uneconomic and supersedes the use value of land. The absence of any integrated rural land use policy only worsens this disintegration process. While an agricultural land protection law is undoubtedly long overdue, its densification component needs to be carefully revisited to address the imminent uncertainties with agricultural practices and the ongoing devaluation of rural land as in fact the villages are suffering from the ‘young men do not want to farm anymore’ syndrome (Wilk 2006).

Despite the fact that the new land reform measure is unfamiliar to the villages of Bangladesh, the application of density-design in developing contexts can go flawed by a range of perceived uncertainties as cautioned by Jenks (2000). This paper with leanings from the empirical context of Bangladesh through studio exercise, consultation with villagers and the secondary statistical data also cautions that the uncertainties exacerbate through the changing rural settlements dynamics. Therefore, the sustainability gains from densification in the villages of Bangladesh will depend on how well the densification strategies are able to address the disintegrating dynamics among the rural society, traditional agricultural practice and the land. Further insights on the prospects of a practicable densification strategy are envisaged in the following paragraphs.

No matter how creative the land reform strategies are, the prospect of the effort in securing the rural agricultural land will firstly depend on how well the value of agricultural practices can be restored and continued as a viable and lucrative economic practice for the majority villagers. Softer interventions, i.e., education, training and other empowerment incentives should aim at developing and nurturing positive attitude towards villages and farming occupations, state-of-art agro-knowledge-base and the necessary skill-sets for the rural population to integrate with farming practices. Restoring a positive agro-based mindset among the rural populations can protect the rural farmland from its speculative disposals (i.e., land-sale and conversion of land use). Commitment in implementing the detail regulations and enforcements should further suffice such social restoration process.

Secondly, every measure taken for physical restructuring of the rural land should be sensitive enough to promote agricultural practices directly or indirectly. As revealed from the design exercise that the provision of efficient infrastructure services is a prerequisite towards compact development, however, cautions should be taken so that the non-farm land-economy is not stimulated. Non-farm land uses to the farmland must not be permitted. However, it is even more necessary to decide the compatibility of non-farm uses that are invited in villages. Non-farm uses (i.e., industries) should conform to the nature and capacity of the agricultural production system of that particular rural setting so that together the protection of arable land and the better management of non-farm land uses—the densification strategy as a whole can render the villages as the engines of growth and harmonize with the regional setting.

Thirdly, emphasis has to be placed on looking for an intelligent rural form that creates a renewed rural–urban interdependency. Therefore, a more agro-market responsive rural to regional linkage has to be established to redistribute the agrarian labor force. Density design has to incorporate selective mix of non-farm uses in villages with authentic non-agrarian demand and should complement to those in the nearest growth or urban centers. Due to the availability of no-farm jobs within an integrated agro-economic system, the rural society may avoid the depopulation syndrome prevalent among the rural marginal, thereby reducing excessive dependency on urban centers. Within such a reciprocal land use distribution, the rural and urban will not be rivalry geographic entities but sustain and grow together.

Nevertheless, it is inevitable that the agrarian social structure will be constantly influenced by the globalization driven changes and urban expansion (i.e., changes in lifestyle, family structure, livelihood options, etc.). The true prospect of densification will be relied on how the spatial structures of the rural system fill the gaps created in the social structure with explicit consideration of the carrying capacity of the given rural system to the extent that the characteristics of the ‘rurality’ is not jeopardized. The southern perspectives on the settlements dynamics would help to recognize those gaps within the degraded human-land relationship than blindfolded by globalization and its western progenies.

The paper confirms that because of the ongoing structural and spatial changes of the present day rural settlements similar to many of the developing countries, more context specific strategiec opportunities should be sought after to cope with the evolving settlements dynamics. While there is no harm to employ the idea of rural densification, however to qualify itself as a feasible planning tool, it must have to satisfy the dynamics that influence the ongoing socio-spatial changes of villages in Bangladesh. No matter how innovative the proposed development control appears to be, it has to be realistic, hence adaptable for the rural communities not only to stop any indiscriminate use of land but also sustain growth.

## Conclusions

The 21st century villages in the developing context of Bangladesh are in the constant process of convergence with the urban. Increasing rural–urban linkages, accessibility to non-farm jobs and urban lifestyle reduce the differences in social-cultural practices thereby the spaces are reconfigured to fit the earlier ones. However, the majority idealization of the ‘rural’ is conveyed more by emotion than rationales and the changes are left unappreciated. Therefore, it is no surprise that, decision making for the villages is governed by the biased mental construction of the rural; thereby fails to correspond to the contextual dynamics allowing intrusion of conflicting strategies.

Given the present-day unwanted changes in the rural land use and multiplicity in depletion of the rural resource base, government’s attempt towards the agricultural land protection and land use bill may have some prospect. But for that, the two-fold objectives of the densification component need to be adequately realized in relation to the recognition of the settlements dynamics that are imbuing a subtle separation between the villagers and their land. Densification as a land use tool takes control of the physical redistribution of the agricultural and non-farm spaces. However, its real prospect will depend on how well it re-cultivates the value of agricultural practices in the diminishing agrarian mindset among villagers.

Contextual exploration of the rural settlements dynamics reveals that the dynamics are neither similar to those prevalent in the western context. Hence, there is an emerging need to build locally situated knowledge for any land regulation measure (i.e., densification) to be successful. In fact, this the time to ‘think universally, see globally, behave regionally, act locally but insightfully’ (Singh 2011a, p. 130) to look in retrospect to the other south and south-east Asian cases that face similar problems of delinking the rural population from their traditional agro-livelihoods and how they are building their own knowledge (i.e., Cleary and Eaton 1996; Rigg 2006; Singh 2011b). Worthy to mention, many of these Southeast Asian rural societies had their common colonization history, from where new postcolonial rural geography with authentic southern perspectives can emerge.

To conclude, given the year on year decreasing per capita land parcel in rural areas, the need for a land reform strategy cannot be ignored. With increasing contestation of rural settlements dynamics within the fast changing globalizing world, the paradox of a sustainable rural future will depend on the sensitivity to recognize the context-specific dynamics that drive the socio-spatial changes. The dynamics should be mutually inclusive towards the processes of densification or any other creative land reform strategy. Failure in doing so could render the villages victim of the bias created either by our romantic ideation about the ‘rural’ or the hegemony of knowledge we often borrowed from the West.

## Footnotes

1 At least two years’ imprisonment and penalty of BDT 50,000/- to BDT 10 lac have been proposed in case of violation of the law (BSS 2011).

2 During the 40 years’ time-span (1961-2001), urban centers in Bangladesh have increased from 78 to 531 (IMF 2013).

3 The functionally landless households (possessing up to 0.49 acre of land, yet too small to economically feasible farming) grew from 60.5 % to 65.1 % in 2005–2010 (BBS 2010).

4 The area of forest cover decreased from 17.5 % to 15.1 % during 2002–2008 (BBS 2009a).

5 Average 1 % loss of cultivable land annually (Bangladesh Planning Commission 2009).

6 Farmland per household reduced from 1.50 to 1.20 acres in 1996–2005 (Quasem 2011).

7 Agricultural households decreased to 34.90 % in 2008 from 39.77 % during 1983–84 (BBS 2009b).

8 Percentage share of income of households from agriculture decreased from 40.1 to 29.73 in 1991–2010 (BBS 2011).

9 During 1975–2000, the area of low-lift pump irrigation and high yielding variety rice cultivation have increased three fold and the use of chemical fertilizers per ha. has been doubled (Ali 2007, p. 725). In addition, studies show that small farmers are more likely to use power tillers as they can not afford feeding the cattle (Asaduzzaman 2002).

10 During 1975–1995, salinity level of soil increased from 1.5 to 2.5 million hectares (Ramamasy and Baas 2007). This has been doubled by the year 2007 (Harun-ur-Rashid and Islam 2007).

11 The area designated for shrimp farming has been expanded from 64,000 to 140,000 hectares (Economic Adviser's Wing 2012), p. 92.

12 Average number of fragments of rural land per hectare increased from 3.03 in 1983 to 3.57 in 1996 (Saha 2002, p 48).

13 Housing occupies 78 % of the total converted land, sometimes 89 % in peri-urban villages (Quasem 2011).

14 During 1983 and 2008, the average per capita arable land declined from 2.27 to 1.01 acre indicating growing landlessness (Uddin and Haque 2011, p. 16).

15 ‘Khas’ in Bengali means abandoned private land now vested to government, 35.7 % of ‘khas’ lands are illegally occupied (Barakat et al. 2001).

16 Literacy rate in rural areas has increased from 21.2 to 44.7 % during 1991–2011 (BBS 2012, p xiii).

17 Flow of remittances emerged as dominant factors in household dynamics increasing household income from 10.6 % in 1991–92 to 17.28 % in 2010 (BSS 2011, p 32).

18 Households with access to electricity in rural areas increased from 31.19 to 42.49 % in 2005–2010. Over 56.7 % household in rural area mobile phone in 2010 as against 6.05 % in 2005 (BSS 2011, p 24).

19 About 39.07 % villagers enjoy the cable TV, which was only 14.92 % in 2004 (BBS 2012, p xi).
